# Transcriptomic profiling of adipose tissue inflammation, remodeling, and lipid metabolism in periparturient dairy cows (*Bos taurus*)

**DOI:** 10.1186/s12864-020-07235-0

**Published:** 2020-11-23

**Authors:** David Salcedo-Tacuma, Jair Parales-Giron, Crystal Prom, Miguel Chirivi, Juliana Laguna, Adam L. Lock, G. Andres Contreras

**Affiliations:** 1 Department of Physiology, College of Natural Sciences, Michigan State University, East Lansing, MI 48824 USA; 2 Department of Animal Science, College of Agriculture and Natural Resources, Michigan State University, East Lansing, MI 48824 USA; 3 Department of Large Animal Clinical Sciences, College of Veterinary Medicine, Michigan State University, East Lansing, MI 48824 USA

**Keywords:** Periparturient period, Lipogenesis, Lipolysis, Adipose tissue inflammation

## Abstract

**Background:**

Periparturient cows release fatty acid reserves from adipose tissue (AT) through lipolysis in response to the negative energy balance induced by physiological changes related to parturition and the onset of lactation. However, lipolysis causes inflammation and structural remodeling in AT that in excess predisposes cows to disease. The objective of this study was to determine the effects of the periparturient period on the transcriptomic profile of AT using NGS RNAseq.

**Results:**

Subcutaneous AT samples were collected from Holstein cows (*n* = 12) at 11 ± 3.6 d before calving date (PreP) and at 6 ± 1d (PP1) and 13 ± 1.4d (PP2) after parturition. Differential expression analyses showed 1946 and 1524 DEG at PP1 and PP2, respectively, compared to PreP. Functional Enrichment Analysis revealed functions grouped in categories such as lipid metabolism, molecular transport, energy production, inflammation, and free radical scavenging to be affected by parturition and the onset of lactation (FDR < 0.05). Inflammation related genes such as *TLR4* and *IL6* were categorized as upstream lipolysis triggers. In contrast, *FASN, ELOVL6, ACLS1,* and *THRSP* were identified as upstream inhibitors of lipid synthesis. Complement (*C3*), *CXCL2*, and *HMOX1* were defined as links between inflammatory pathways and those involved in the generation of reactive oxygen species.

**Conclusions:**

Results offer a comprehensive characterization of gene expression dynamics in periparturient AT, identify upstream regulators of AT function, and demonstrate complex interactions between lipid mobilization, inflammation, extracellular matrix remodeling, and redox signaling in the adipose organ.

**Supplementary Information:**

**Supplementary information** accompanies this paper at 10.1186/s12864-020-07235-0.

## Background

The transition from late pregnancy to early lactation (the periparturient period, PPE) represents a major metabolic challenge to mammals and is dependent on an extensive series of physiological adaptations that include many, perhaps most, body tissues and involve all nutrient classes [1, 2]. The PPE is especially challenging in dairy cows as these animals have increased energy requirements driven by fetal growth and copious milk production. At the same time, cows have an inadequate appetite and feed intake to meet the energetic costs of maintenance plus pregnancy or lactation, resulting in a state of negative energy balance (NEB). This energetic deficit depletes liver glycogen stores and increases the use of amino acids and fatty acids (FA) as energy sources [3]. Throughout the PPE, the primary source of FA is lipolysis in adipose tissues (AT) [4]. Cows successfully adapt to NEB when the release of FA from AT is limited to concentrations that can be fully metabolized for energy needs [4]. However, when lipolysis is excessive, cows exhibit elevated levels of plasma FA around parturition that are associated with increased disease susceptibility and limited milk production [5].

The AT adapts to support the energy needs of the PPE by increasing its responsiveness to lipolytic stimuli. At the same time, adipocytes, the essential cellular component of AT, become resistant to the anti-lipolytic effects of insulin. In addition to driving the release of FA and glycerol from adipocytes, AT lipolysis also involves a remodeling process characterized by an inflammatory response with immune cell infiltration composed mainly of macrophages [6]. These mononuclear cells are the predominant immune cell type in AT of ruminants [7]. In dairy cows, when lipolysis is severe, AT macrophages make up 20% of the cells in the stromal vascular fraction (i.e., non-adipocytes) or 2% of the total number of cells in the AT [8, 9]. Although AT physiological adaptations to NEB during PPE are well characterized, the transcriptomic mechanisms that govern these changes are still poorly understood.

Here we report the results of a next-generation RNAseq study in subcutaneous AT collected at three different time points during the PPE. Parturition and the onset of lactation induce profound transcriptomic changes in genes with functions grouped by in silico analysis in categories such as lipid metabolism, molecular transport, energy production, inflammation, extracellular matrix structure, and free radical scavenging. These results offer a comprehensive characterization of gene expression dynamics in periparturient AT and demonstrate the close interactions between lipid mobilization, inflammation, and redox signaling in the adipose organ.

## Methods

### Animal model

Twelve healthy multiparous Holstein cows at the Michigan State University Dairy Field Laboratory were used for this longitudinal cohort study. At the moment of selection, cows were non-lactating and pregnant (210–240 days of gestation). The body condition score (BCS) was assessed weekly by three trained technicians, and the average score was calculated [10]. Cows were blocked (six blocks) by the last BCS measurement before parturition (up to 0.50-unit difference using the scale of 1 = thin and 5 = fat in 0.25 increments), previous lactation 305-d mature-equivalent yield (MEq; within 5700 kg), and parity (up to 1 lactation difference). The values (mean ± SD) for BCS, MEq and parity were 3.53 ± 0.22, 32,182 ± 3752 kg, and 2.67 ± 0.65 respectively. Cows were housed in tie-stalls bedded with sawdust. All animals received a close-up (− 21 d pre-calving to parturition) and a fresh (1–15 d in lactation) diet that were formulated to meet or exceed their nutritional requirements according to NRC (Supplement Table 1, [11]). None of the 12 cows had a health event reported during the study and remained in the herd after experiments.

### Sample collection

Blood samples were collected weekly from 3 wk. before the expected parturition date until 3 wk. after calving. Blood was drawn before the morning feeding via coccygeal venipuncture using coated collection tubes (K_2_ EDTA), centrifuged for 20 min at 3000×g (15 °C) for plasma fraction collection, and then stored at − 80 °C until further analysis. Plasma concentrations of insulin, glucose, free FA (FFA), and β-hydroxybutyrate (BHB) were determined using an Olympus AU640e chemistry analyzer (Olympus America, Center Valley, PA, USA) at the Michigan State University Veterinary Diagnostic Laboratory (Lansing, MI, USA).

Subcutaneous AT (SCAT) samples were obtained from the right flank at 11 ± 3.6 d before the expected calving date (PreP) and 6 ± 1 d (PP1) and 13 ± 1.4 d (PP2) after parturition, using the surgical procedure described by Mann et al. [12], with modifications detailed in [13]. The site of the incision was moved 3–4 cm at each collection timepoint. Five grams of SCAT were collected, snap-frozen in liquid nitrogen, and stored at − 80 °C for RNA extraction. A subsample was fixed in formaldehyde for 12 h for histological analysis. The skin was closed using a continuous interlocking suture with Braunamid (USP1, Aesculap, Center Valley, PA, USA). Sutures were removed 12–14 d after each procedure.

Histological analyses were performed using hematoxylin- and eosin-stained sections from paraformaldehyde fixed paraffin-embedded tissue. The area of adipocytes in 5 randomly selected fields per section was measured using the Adiposoft plugin (v. 1.15) for ImageJ Fiji (v 2.0.0), as described in [14].

### RNA seq analyses

Total RNA was extracted from frozen samples using Trizol and the Quick RNA MiniPrep kit (R1054; Zymo Research, Irving, CA, USA) that includes a DNase step to remove genomic DNA according to the manufacturer’s protocol. Purity, concentration, and integrity of mRNA were checked using a NanoDrop 1000 spectrophotometer (Thermo Scientific, Wilmington, DE, USA) and an Agilent Bioanalyzer 2100 system (Agilent Technologies, Santa Clara, CA, USA). All samples had a 260:280 nm ratio between 1.9 and 2.1 and RNA integrity number ≥ 8. Samples were sent to Novogene Corporation Inc. (Sacramento, CA, USA) for sequencing in Illumina platform. Data quality control was performed with FastQC v.0.11 (www.bioinformatics.babraham.ac.uk/projects/fastqc/). After data filtering, clean reads were mapped to the bosTau7 reference genome using HISAT 2.1.0 [15]. The average mapping ratio with the reference genome was 95.06%. After genome mapping, HTseq v.0.6.1 was used to count the number of reads to perform differential expression analysis (Supplement Table 2, [16]).

Gene count matrix was uploaded to the NetworkAnalyst 3.0 platform and filtered for genes with low transcription abundance (less than 3 reads per gene in all the samples), and features with constant values (either 0 or empty) were removed [17]. The gene counts were then normalized using log2. Principal components analysis (PCA) and 3D PCA analyses were plotted with R v3.6.1 packages. The R package edgeR v3.4.2 was used to detect differentially expressed genes in pairwise comparisons and differences among time points [18]. Genes with fold changes > 1, and false discovery rates (FDRs) < 0.05 were defined as Differential Expressed Genes (DEGs) and captured for further analysis. All data is available in NCBI Gene Expression Omnibus (accession number: GSE159224).

### Enrichment analysis and networks of gene interaction

Expression analyses were imported into the Ingenuity Pathways Analysis (IPA) Software (Ingenuity Systems Inc., NY, USA) and Metascape for functional enrichment analysis [19]. IPA uses information from databases to predict regulatory networks associated with an expression list of genes and determines a statistical Z-score for each network. This Z-score predicts how the network is altered as the result of the gene expression profile given. Canonical pathways and functional regulatory networks of upstream regulators were identified by the prediction algorithms and the hypergeometric distribution algorithm. The significance for pathway analyses was set at *P* < 0.05, and that of networks was set at *P* < 0.01. Irrelevant diseases and processes specific to other species were removed.

### Statistical analysis

The study’s sample size is based on previous PPE studies by our group, where we determined the impact of parturition and the onset of lactation on the expression of specific genes using targeted gene expression analyses [13, 20, 21]. All individual data for BW, BCS, and blood metabolites were averaged per period (PreP, PP1, and PP2) and analyzed using the MIXED procedure of SAS version 9.2 (SAS Institute Inc., Cary, NC). The model used was Yijklm = μ + Bi + C(BiFk)j + Fk + Jm + eijklm, where Yijklm is the dependent variable, μ = overall mean; Bi = random effect of block; C(BiFk)j = random effect of cow within block and period; Fk = fixed effect of period; Jm = random effect of Julian date; and eijklm = residual error. NEFA values were logarithmically transformed to achieve normal distribution. Pairwise mean comparisons evaluated significant effects, and *P*-values were adjusted for multiple comparisons using the Tukey-Kramer method. Friedman’s nonparametric test was used for analyzing BHB concentrations due to non-normal distribution. Significance was declared at *P* ≤ 0.05.

## Results

### Periparturient lipolysis

**Fig. 1:**
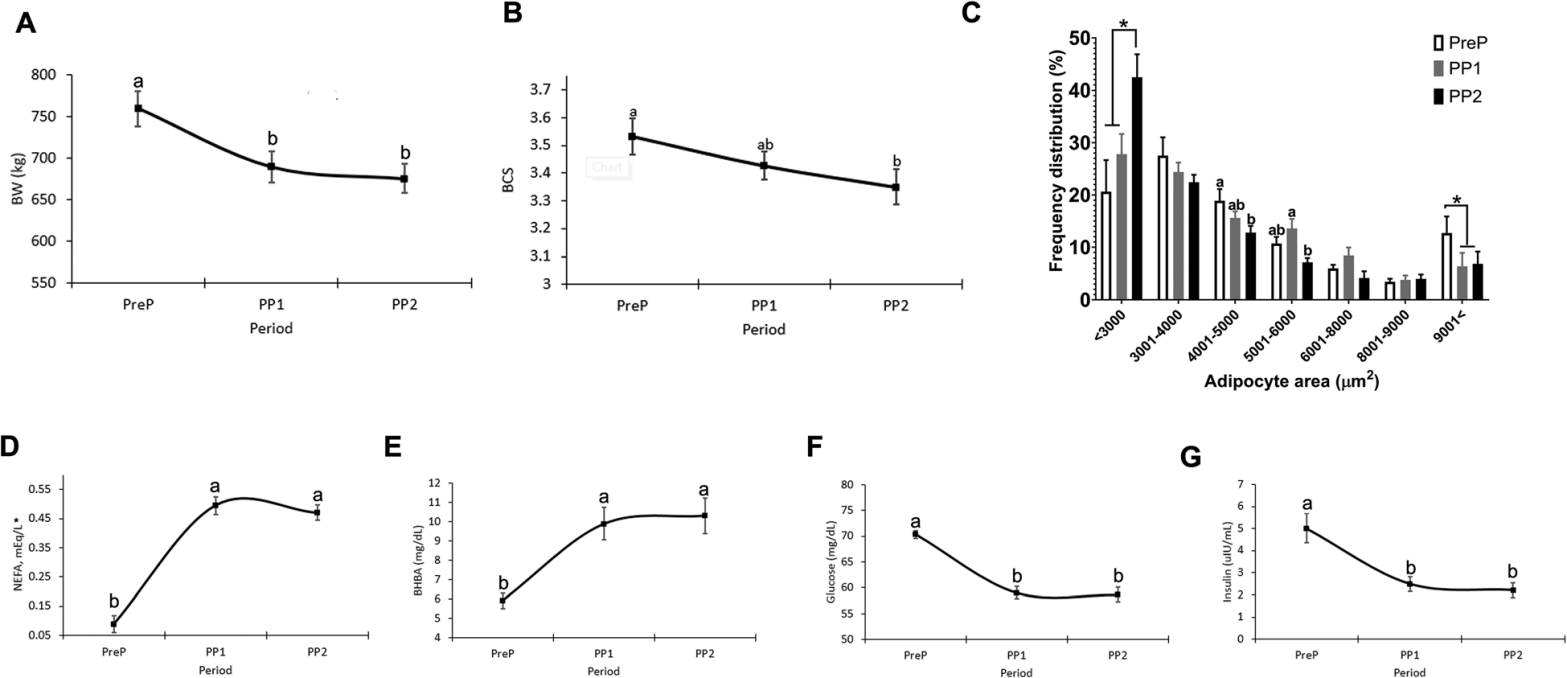
Lipolysis increases postpartum and reduces adipose mass and adipocyte size. Blood and adipose tissue samples were collected from Holstein cows (*n* = 12) at 11 ± 3.6 d before calving date (PreP) and at 6 ± 1d (PP1) and 13 ± 1.4d (PP2) after parturition. **a** Body weight; **b** Body condition score (BCS); **c** Frequency of adipocyte sizes in subcutaneous adipose tissues; **d** Circulating free fatty acids (NEFA); **e** circulating beta-hydroxybutyrate (BHB); **f** Blood glucose and **g** Insulin. Data are means ± SEM or * median ± SEM. Significant differences are indicated by * and letters a, b, c (*P* < 0.05)

FA reserves stored as triglycerides in adipocytes are mobilized by lipolysis to support the energy needs of parturition and lactation during a time of limited feed intake. FA mobilization in this group of cows was reflected in different parameters. First, there was a reduction in cows’ BW and BCS at PP1 and PP2 compared with PreP (Fig. 1a and b). Depletion of triglyceride stores in adipocytes reduced their average size postpartum (PreP = 3951 ± 336 μm^2^; PP1 = 3534 ± 336 μm^2^; PP2 = 3111 ± 336 μm^2^; *P* < 0.05). Lipolysis increased the percentage of smaller (< 3000 μm^2^) and reduced that of the largest (9000 μm^2^) adipocytes at PP1 and PP2 compared to prepartum values (Fig. 1c). Plasma concentrations of FFA and BHB increased after parturition, also reflecting the mobilization of AT lipid stores (Fig. 1d and e). The high lipolysis rate at PP1 and PP2 coincided with a reduction in blood glucose and insulin compared with prepartum values (Fig. 1f and g).

### The effect of parturition and onset of lactation on AT transcription profile

**Fig. 2:**
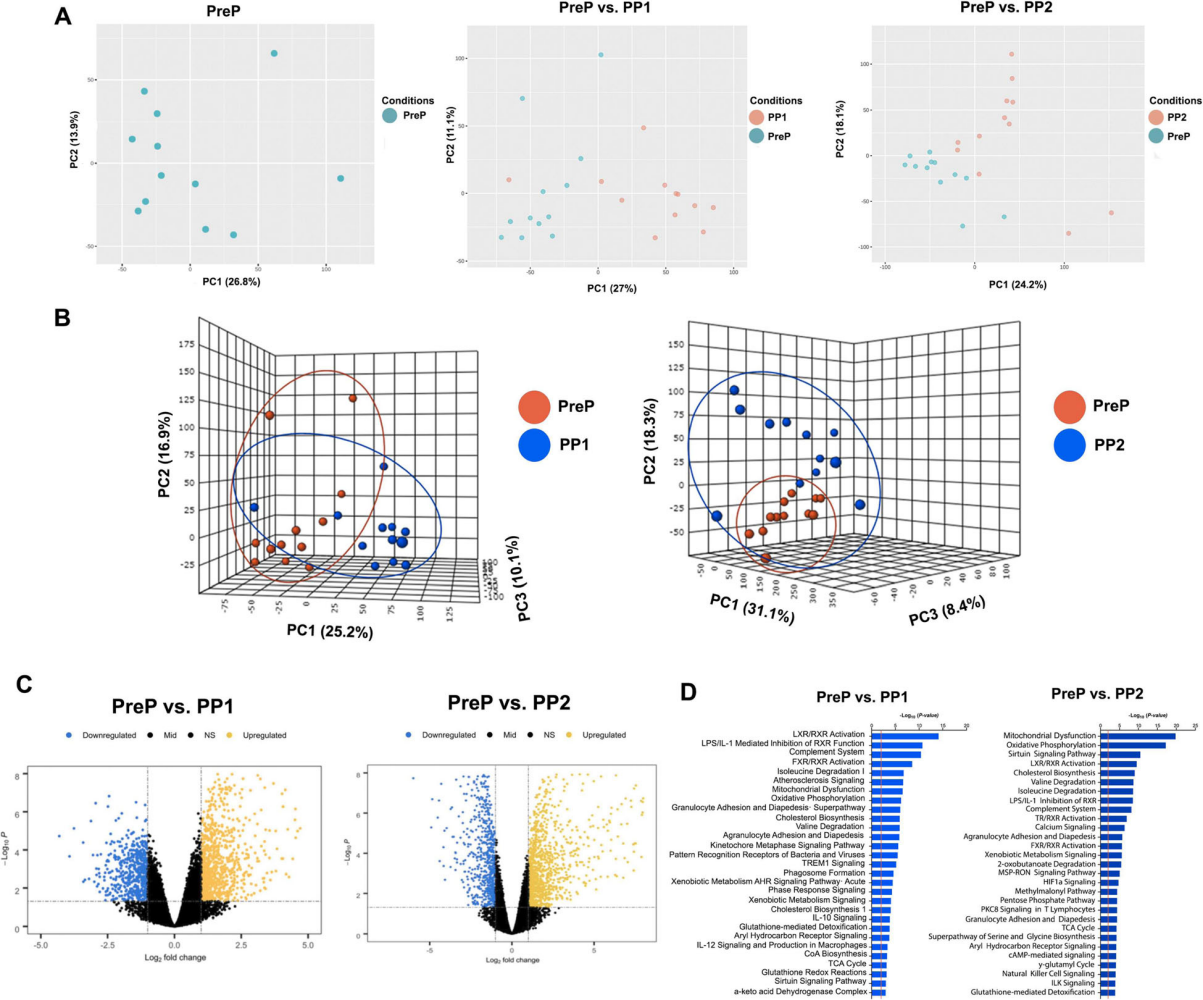
The transcriptomic profile of subcutaneous adipose tissue is altered after parturition and the onset of lactation. Adipose tissue samples were collected from Holstein cows (n = 12) at 11 ± 3.6 d before calving date (PreP) and at 6 ± 1d (PP1) and 13 ± 1.4d (PP2) after parturition. **a** 2D and **b** 3D Principal components analysis (PCA) of adipose tissue transcriptomics data at PreP, PreP vs. PP1, and PreP vs. PP2. **c** Volcano plots of differentially expressed genes at PP1 and PP2 when compared to PreP samples. **d** Enrichment of differentially expressed genes (DEG) analyzed based on canonical pathways categories using Ingenuity Pathway Analysis (IPA). The x-axis indicates the –log10(*p*-value) and red line indicates a threshold of *p* = 0.05 [−log10(0.05) =1.3]

To establish an AT transcriptional profile baseline before parturition, we performed a principal component analysis (PCA) on PreP samples. PCA patterns at PreP demonstrate no clear separation of the samples (Fig. 2a). To determine the effects of parturition and the onset of lactation on AT transcription patterns, we compared PreP to PP1 and PP2. The samples’ separation and composition patterns in 2D and 3D PCA analyses show a clear difference among PreP, PP1, and PP2 (Fig. 2a and b). Differential expression analysis of samples at PreP vs. PP1 identified 1524 DEGs. Of these genes, 921 were upregulated and 603 downregulated (Fig. 2c, Supplement Table 2). The progression of lactation accentuates the changes in AT transcription patterns. When comparing PreP and PP2, a total of 1946 genes were DEG. The expression of 1213 of these genes was upregulated, and that of 733 was downregulated (Fig. 2c, Supplement Table 2). Next, Functional Enrichment Analysis was performed on PP1 and PP2 using Ingenuity pathways IPA and Metascape. As expected, functions grouped in categories such as lipid metabolism, molecular transport, energy production, cell signaling, and free radical scavenging were affected by parturition and the progression of lactation (Fig. 2d).

### Enrichment of gene networks of AT lipid metabolism, inflammation, and remodeling

**Fig. 3:**
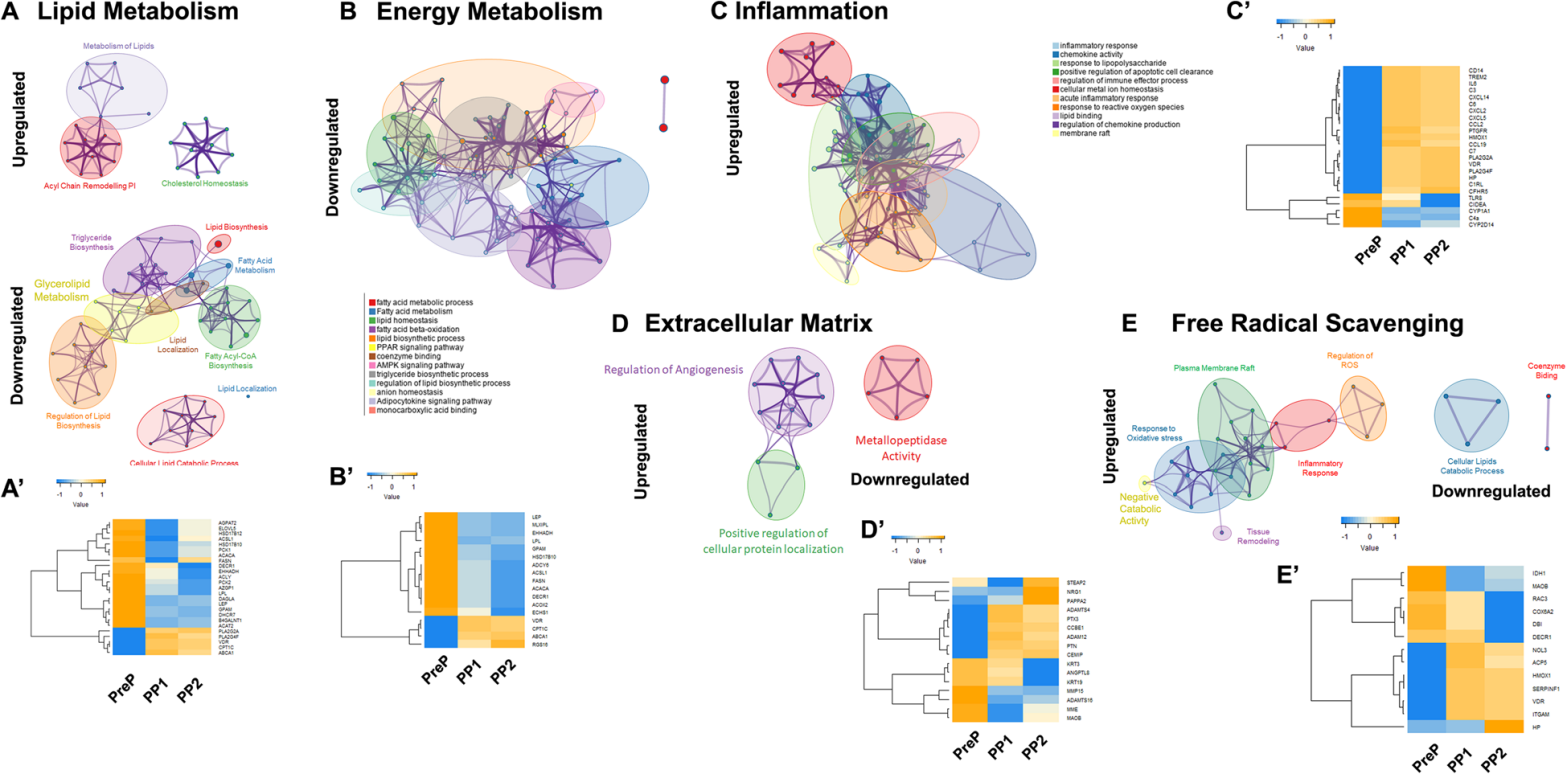
Essential gene networks clusters in adipose tissue of dairy cows are altered after parturition and the onset of lactation. Enrichment network visualization through Metascape showing the functional cluster similarities of enriched terms for differentially up and downregulated genes. Each network matches a heatmap of the differential expressed genes included in the networks. (A, A’) Lipid Metabolism; (B, B′) Energy metabolism; (C, C′) Inflammation; (D, D’) Extracellular matrix; (E, E’) Free Radical Scavenging. (FDR < 0.01). Cluster annotations are color-coded

Since there were more transcriptional changes identified at PP2 compared to PP1 and the majority overlapped between the two timepoints, we selected PP2 DEG and performed enrichment analysis using Metascape and IPA. Results demonstrated enrichment of processes related to lipid metabolism, energy production, inflammation, extracellular matrix remodeling, and free radical scavenging. Few lipid metabolism enriched processes were upregulated at PP2, and these belonged to cholesterol homeostasis and the acyl chain remodeling pathway (Fig. 3A). In contrast, there were several gene networks downregulated at PP2 that included synthesis of FA, FA and glycerolipid metabolism, and lipid and triglyceride biosynthesis (Fig. 3A). Many of these lipid-related downregulated processes are also in the energy metabolism ontology networks, including adipokine signaling and monocarboxylic acid binding (Fig. 3B). There were four expression patterns of lipid metabolism-related genes (Fig. 3A‘). Genes involved in de novo lipogenesis such as *AGPAT2* and *FASN* were downregulated (− 3.3 and − 6.0 Fold Change [FC]) at PP1 compared to PreP, and then their expression started to increase by PP2 with values of − 1.9 FC for *AGPAT2* and − 2.4 FC for *FASN* (Supplement Table 3). The second group of genes exhibited a gradual reduction of their FC expression from PP1 to PP2 compared to PreP. This group of genes belongs to processes related to energy generation and catabolism, including *ACLY* (PP1 = -3.2 FC; PP2 = -2.0 FC)*, LPL* (PP1 = -2.8 FC; PP2 = -1.7 FC), and *DECR1* (PP1 = -2.8 FC; PP2 = -1.9 FC). The third group of genes showed a continuous downregulation at PP1 and PP2, and these included leptin (*LEP;* PP1 = -2.8 FC; PP2 = -1.8 FC), *GPAM* (PP1 = -4.8 FC; PP2 = -2.9 FC), and *ACAT2* (PP1 = -1.7 FC; PP2 = -1.4 FC). The final group had a drastic increase in expression upon parturition. These genes modulate intracellular lipid fluxes, including cholesterol transport *ABCA1* (PP1 = 2.2 FC; PP2 = 1.8 FC), mitochondrial CoA transport *CPT1c* (PP1 = 1.8 FC; PP2 = 1.7 FC), and the vitamin D receptor *VDR* (PP1 = 3.0 FC; PP2 = 1.9 FC). Among the DEGs involved in energy metabolism, two expression patterns were identified (Fig. 3B‘). A group with downregulation below − 4 FC after calving included anabolic genes belonging to the de novo lipogenesis pathway (e.g. *FASN, ACACA*) and a group with permanent upregulation after calving including *RGS16* a regulator of G protein signaling (Fig. 3B‘and Supplement Table 3).

High lipolysis rates induce inflammatory responses within AT in the first 2–3 weeks after parturition [20]. Accordingly, several inflammation-related networks became activated at PP2, including chemokine activity, apoptosis, lipid binding, response to lipopolysaccharide, and membrane rafts (Fig. 3C). DEG belonging to these upregulated networks at PP1 and PP2 included those encoding for pro-inflammatory cytokines (*CCL2, CCL19, CXCL2, CXCL5, IL6)*, macrophage phenotype markers (*CD14, TREM2*), complement (*C3, C6, C7*), and the free heme processing proteins *HMOX1* and *HP* (Fig. 3C‘and Supplement Table 3). DEG that exhibited downregulation at PP1 and PP2 included the gene encoding for the complement component *C4a* and those transcribing cytochrome P450 enzymes *CYP2D14* and *CYP1A1.* Toll-like receptor 8 (*TLR8*) showed a unique expression pattern exhibiting strong downregulation at PP2 compared to PreP and PP1 (Fig. 3C‘and Supplement Table 3).

Due to the rapid reduction in AT mass during the first three weeks after parturition (> 20%, [22]), the AT exhibits considerable changes in its extracellular matrix (ECM) structure gene networks with upregulation of angiogenesis and cellular protein localization and downregulation of metallopeptidase activity (Fig. 3D). Reflecting on the complex regulation of ECM, certain metalloproteases that promote angiogenesis and adipogenesis were upregulated (*ADAMTS4*, *PAPPA2*). In contrast, others such as *ADAMTS16* were downregulated together with specific ECM proteins like keratins 3 and 19 encoded by *KRT3* and *KRT19* (Fig. 3D‘and Supplement Table 3).

Rapid mobilization and oxidation of FA in adipocytes during the PPE trigger intense responses to oxidative stress and reactive oxygen species generation. Accordingly, gene networks that link inflammation and oxidative stress were upregulated, while those that involve catabolism of lipids were downregulated (Fig. 3E and Supplement Table 3). Noticeably, genes associated with heme processing such as *HP* and *HMOX1* were upregulated after calving (Fig. 3E‘and Supplement Table 3). While *IDH1*, an NADPH producing enzyme, and *MAOB*, an amine oxidase, were downregulated (Fig. 3E‘and Supplement Table 3).

### Pathway analyses of AT lipid metabolism, inflammation, and remodeling

**Fig. 4:**
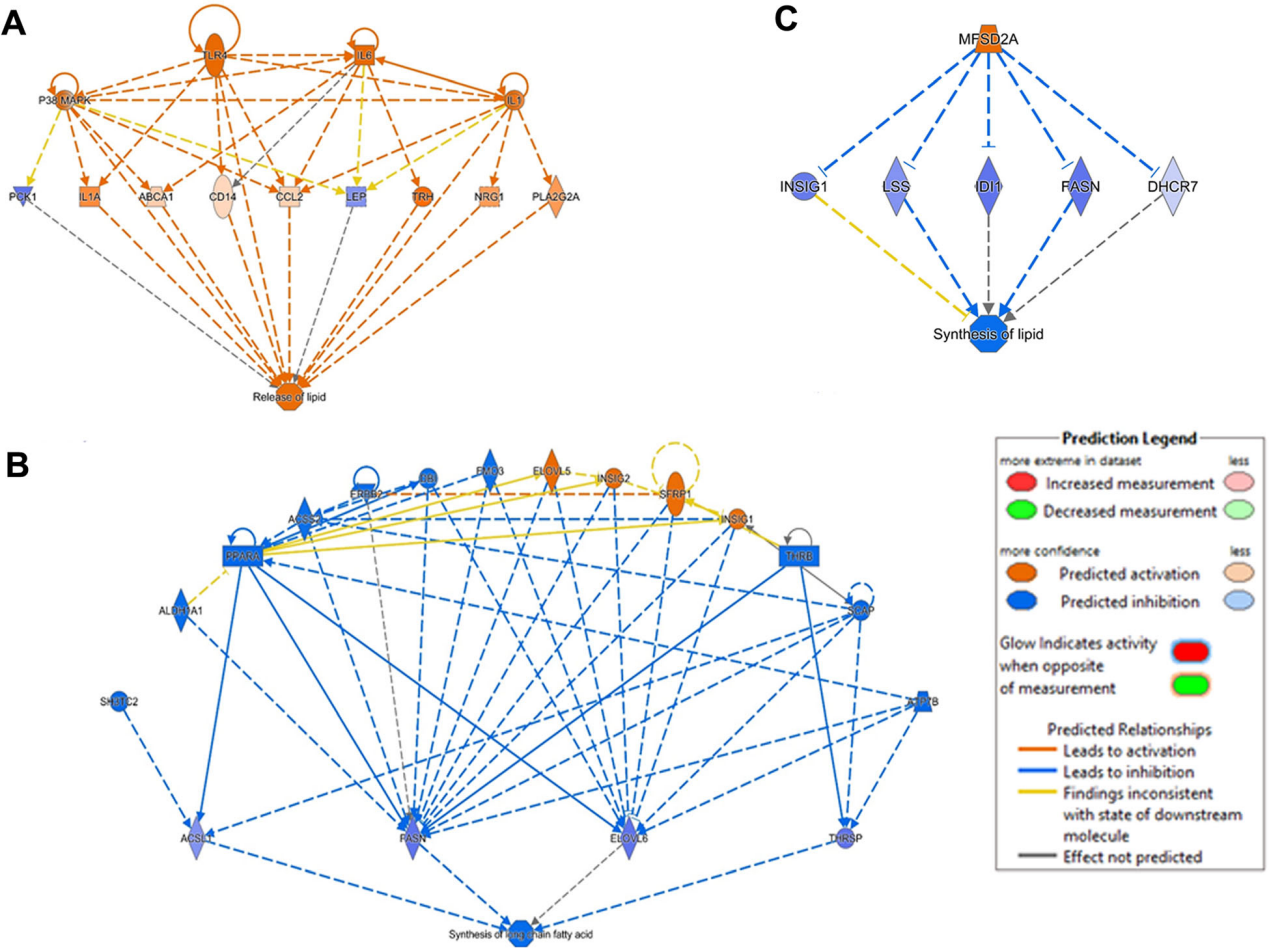
Impact of transcriptional profile changes after parturition and the onset of lactation on upstream regulators and biological functions as predicted by IPA analysis. Network analyses combined differentially expressed genes with upstream regulators and physiological functions to identify factors related to the activation or inactivation of lipid mobilization biological processes (*p* ≤ 0.05). After parturition, hubs in the networks colored orange are predicted to have activation, and those in blue are predicted to be inhibited. Color lines connecting hubs indicate IPA prediction of hubs whose activity lead to upregulation (orange line) or downregulation (blue line). Networks analyzed: (**a**) Release of lipids; (**b**) Inhibition of long-chain fatty acids synthesis; (**c**) Inhibition of lipid synthesis of lipids

**Fig. 5:**
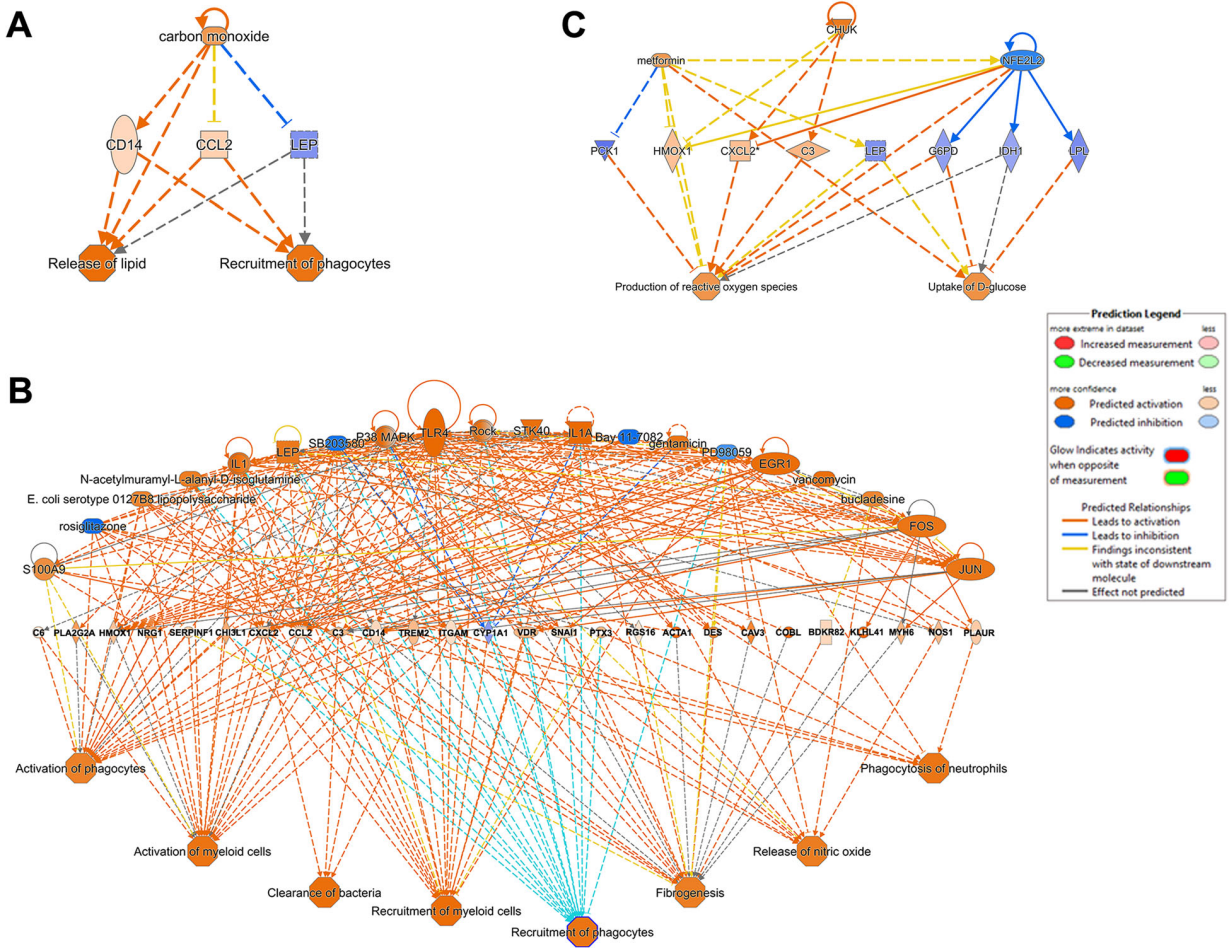
Impact of transcriptional profile changes after parturition and the onset of lactation on upstream regulators and biological functions as predicted by IPA analysis. Network analysis combined differentially expressed genes with upstream regulators and biological functions to identify factors related to the activation or inactivation of lipid mobilization biological processes (p ≤ 0.05). After parturition, hubs in the network colored orange are predicted to have activation, and those in blue are predicted to be inhibited. Color lines connecting hubs indicate IPA prediction of hubs whose activity lead to upregulation (orange line) or downregulation (blue line). Networks analyzed: (**a**) Activation of phagocytes recruitment and lipid release; (**b**) Activation of immune response; (**c**) Production of ROS and glucose uptake

Ingenuity pathway analyses determined upstream regulators related to DEG identified at PP2. The activation of inflammatory signals, including P38 MAPK, TLR4, IL1, and IL6 were identified as triggers of lipolytic activity (Fig. 4a). In contrast, several components of lipogenic pathways acted as inhibitors of *FASN*, *ELOVL6*, *ACLS1*, and *THRSP* and thus reduced the synthesis of lipids (Fig. 4b). The gene encoding primary facilitator superfamily domain-containing protein 2a (*MFSD2A*) also suppressed lipogenic activity (Fig. 4c). As expected, the regulation of inflammatory responses within AT during the PPE is complex and includes many upstream regulators that are interconnected. Key pathways identified by IPA included activation and recruitment of phagocyte and myeloid cells, phagocytosis, and fibrogenesis (Fig. 5a). Carbon monoxide acted as an upstream regulator of lipolysis and phagocyte recruitment linking inflammation and lipid release (Fig. 5b). Finally, the gene *CHUK,* encoding the inhibitor of NFKβ kinase complex subunit alpha, was identified as a possible promotor of reactive oxygen species production linking inflammation and oxidative stress [23]. Although *CHUK* is only one component of the kinases that phosphorylate NFKβ, its inflammatory activity induces the activation of complement (C3), *CXCL2* encoding macrophage inflammatory protein 2-alpha, and *HMOX1 (*Fig. 5c).

## Discussion

During the PPE, AT reserves of FA support lactation and bodily functions in an NEB environment. Lipolysis makes FA available as an energy substrate to tissues by breaking down triglyceride molecules stored within adipocyte lipid droplets. In this group of cows, a high PPE lipolysis rate was reflected in the reduction of BW and BCS post-calving, increased postpartum plasma NEFA and BHB, and reduced adipocyte size as lactation progressed. These changes occurred during the first 2 weeks of lactation and were accompanied by variations in AT gene expression patterns that were analyzed by measuring their transcript levels using NGS.

To our knowledge, this the first next-generation RNAseq study performed in AT from multiparous PPE cows and the second in PPE dairy cattle after Mellouk et al. [24] who did a similar transcriptomics evaluation in first lactation heifers. In our current study, transcription profiling identified 1524 and 1946 DEG at PP1 and PP2, respectively, compared to PreP. In the report by Mellouk and colleagues, heifers with moderate NEB exhibited 977 DEG at one week post-calving when comparing the transcriptome with that at four weeks before parturition. In both studies, parturition and the onset of lactation coincided with abrupt and intense changes in AT gene expression profiles.

### Periparturient AT lipid and energy metabolism

Enrichment analyses in the present study and that of Mellouck et al. [2020] identified genes related to lipid and energy metabolism processes overly represented as DEG (Fig. 3). This is an expected observation reflecting AT’s rapid response to reduce NEB that is characterized by an inhibition of pathways that promote the synthesis of FA and triglycerides [25]. By diverting FA away from triglyceride formation, the AT prioritizes FA released by lipolysis for export as energy substrates. Previous RT-qPCR and microarray experiments reported the downregulation of lipogenic genes that govern FA synthesis pathways in AT during the PPE, including *FASN* [26–28], *ELOVL6* [26, 29], *PCK1* [30, 31]*, ACACA* [13, 32]*,* and *ACLS1* [32, 33]. Our IPA analysis found these same genes and *ELOVL5, THRSP,* as upstream inhibitors of FA synthesis. An important finding in our analyses is that following parturition, the inhibition of FA synthesis related genes is not static. Genes that have a predominant role in lipogenesis such as *FASN, ELOVL5, ACLS1,* and *PCK1* increase their expression by PP2 responding to the improvement in NEB. Together, our results demonstrate that lipogenesis inhibition is a feature of AT transcriptomic adaptation to the PPE that is reduced as lactation progresses. The gradual reactivation of lipogenesis likely minimizes the release of FA into circulation as NEB diminishes.

Another necessary transcriptional adaptation of AT to the PPE is increasing the capacity of adipocytes to export lipids. Cows in this study exhibited upregulation of the cholesterol homeostasis pathway characterized by higher expression of *ABCA1* (Fig. 3A and A‘)*.* Lipolysis activates cholesterol efflux in adipocytes as the breakdown of lipid droplets releases cholesterol molecules into the cytoplasm [34]. The protein ABCA1 is critical for delivering free intracellular cholesterol and phospholipids to extracellular apolipoproteins and thus activating the transport of lipids from AT [35]. Postpartum activation of the cholesterol homeostasis pathway coincides with the well-characterized plasma cholesterol dynamics in dairy cows that reach their nadir immediately after parturition and rapidly rebound to pre-calving levels by four weeks into lactation [36]. The pattern of AT *ABCA1* transcription observed in the present study coincides with that reported in the liver [36]; thus, both organs activate their cholesterol homeostasis pathways to accommodate the sizeable periparturient flux of lipids into circulation.

Cows in our present study also exhibited, at least at the transcription level, post-calving activation of the acyl chain remodeling pathway (Lands Cycle). Primarily driven by the upregulation of the phospholipases *PLA2G2A* and *PLA2G4F* at PP1 and PP2 (Fig. 3A‘), this pathway regulates the size of lipid droplets and may be necessary for targeting smaller droplets generated during lipolysis to intracellular organelles such as the mitochondria and the rough endoplasmic reticulum [37]. Since mitochondria is an essential target for FA released during lipolysis in adipocytes [38], the upregulation of *CPT1C* at PP1 and PP2 is noteworthy (Fig. 3A‘). This gene encodes an isoform of the rate-limiting enzyme in mitochondrial FA oxidation, carnitine palmitoyltransferase 1. Simultaneously, AT showed downregulation of *LEP,* coinciding and likely explaining the low circulating levels of leptin observed during the PPE [39]. By reducing leptin, the AT exerts an orexigenic effect to counterbalance NEB. Together these transcriptomic changes activate the oxidative machinery of adipocytes preparing these cells for using FA as an energy substrate in the mitochondria and enhance energy intake systemically.

Although lipolytic activity is primarily regulated by the phosphorylation rate of lipases and not by gene transcription patterns [40], it is essential to highlight the drastic postpartum downregulation of *MAOB*. This gene encodes monoamine oxidase B, an enzyme that degrades lipolytic amines such as norepinephrine. The responses of AT to adrenergic stimuli in dairy cows are increased during the first month after parturition compared to the non-lactating period [41]. A limited transcription of *MAOB* provides a mechanistic target for this physiological response, and therefore future studies should examine protein expression and activity patterns of this enzyme around parturition.

### Adipose tissue inflammation

Lipolysis is an inflammatory event that triggers immune cell infiltration and extracellular remodeling in humans [42], rodents [43], and dairy cows [4]. Our study’s enrichment analyses identified several pathways in PP1 and PP2 samples that likely modulate the inflammatory process in AT postpartum (Fig. 3C). Our DEG (Fig. 3C‘) and IPA (Fig. 5b) analyses highlighted the cytokines *IL6*, *CXCL2*, and *CCL2* as upstream targets for pathways involved in the recruitment and activation of phagocytes and phagocytosis. Zhang and colleagues characterized lipolysis induced IL6 secretion as a hormone-sensitive lipase activation-dependent event in rodents [44]. In dairy cows, postpartum AT *IL6* upregulation is a commonly described transcriptomic finding [45]. Both the macrophage inflammatory protein 2-alpha, encoded by *CXCL2,* and monocyte chemoattractant protein-1, encoded by *CCL2,* are promoters of macrophage infiltration into AT [46]. Postpartum upregulation of these genes in AT was reported by our group and others using RT-qPCR approaches [13, 27].

Macrophage infiltration is a significant characteristic of lipolysis induced inflammation in AT [47]. Increased expression of *CD14* postpartum in this group of cows provides further evidence for the enhanced trafficking of macrophages into AT during lipolysis as the CD14 protein is a macrophage marker in cattle [48]. This finding coincides with reports from different research groups demonstrating an increased number of macrophages in AT postpartum, especially in cows with high lipolysis rates using transcriptomics [27], flow cytometry [8], and immunohistochemistry [45, 49]. A new finding of our present study is the identification of *TREM2* as DEG*.* This gene encodes for a transmembrane glycoprotein that binds with apolipoproteins and phospholipids. TREM2 expression is abundant in macrophages with an anti-inflammatory phenotype and is a marker for high cholesterol metabolism and oxidative phosphorylation capacity [50]. Since phenotyping AT macrophages is difficult in bovines, TREM2 expression in PPE cows warrants further investigation as it could represent a new marker for mononuclear cells with metabolic functions also described in the literature as metabolically activated macrophages [51].

The DEG analysis in our study demonstrated a postpartum upregulation of genes encoding the complement proteins C3 and C5. These results align with proteomic studies by Zachut and colleagues identifying the same peptides with a similar enhanced expression pattern postpartum compared to pre-calving timepoints [52]. C3 is the main effector protein of the complement system. Convertases cleave C3 to generate C3a and C3b and C5 to yield C5a and C5b upon activation by the inflammatory process. C3a downstream product C3adesArg (i.e., acylation stimulating protein) and C5a are both potent inhibitors of lipolysis and promoters of triacylglycerol synthesis and glucose transport in adipocytes and muscle cells [53, 54]. C3a and C5a are good examples of how inflammatory mediators released during the resolution stage of inflammation may exert a negative feedback loop that reduces lipolysis in adipocytes.

The IPA analysis results in our study link, for the first time in dairy cattle, the expression of TLR4 and IL6 with lipid release pathways. This connection is well described in human and rodent studies. Activation of TLR4 by endotoxins or FA stimulates the phosphorylation and degradation of IκB proteins via a MyD88-dependent pathway. This leads to the translocation of NF-κB, which triggers the synthesis of pro-inflammatory cytokines, including IL6 and TNFα [55]. The latter promotes lipolysis by impairing the expression and function of perilipin. This causes the thinning of the protein envelope of the lipid droplet, making it more susceptible to HSL’s action [56]. TLR4 can also activate the mitogen-activated protein kinase /extracellular signal-regulated kinase (MEK/ERK) pathway in a MyD88-independent manner. MEK/ERK pathway phosphorylates the beta-adrenergic receptors and PKC, ultimately leading to HSL and perilipin phosphorylation [57, 58]. Additionally, MEK/ERK stimulates TNFα production [59]. Given the high incidence of inflammatory diseases with high levels of circulating endotoxins in PPE cows [60], further research should focus on elucidating the mechanisms that link TLR4 activation with dysregulation of lipolysis.

### Adipose tissue remodeling

Lipolysis is a catabolic process that leads to a drastic reduction in the mass of the AT in PPE cows. As with any remodeling process, this change requires robust proteolytic activity, which in the animals in the present study was reflected in enhanced transcription of metallopeptidase and angiogenesis-related pathways. Our DEG analysis indicated postpartum upregulation of the matrix metalloproteinases *ADAMTS4* and *ADAM12* that are members of ADAM/ADAMTS system. Both genes encode proteins belonging to the disintegrin and metalloproteinases group of proteases. The transcription of *ADAMTS4* produces a homonym enzyme that acts on aggrecan. Although this proteoglycan is more abundant in cartilage, it is also present in AT and is a potent promoter of adipogenesis in preadipocytes. The activity of ADAMTS4 is a determinant in the action of aggrecan on adipocyte progenitor cells. Remarkably ADAMTS4 is abundantly present in periods of AT expansion postnatally [61]. ADAM12 or meltrin alpha is a metalloprotease disintegrin. Similar to ADAMTS4, ADAM12 is a proadipogenic factor, and its metalloprotease activity is required for the differentiation of perivascular preadipocytes into adipocytes [62]. Therefore, the upregulation of this aggrecanase may be an early indicator of the reactivation of adipogenic and lipogenic activity after the lipolysis peak postpartum.

### Adipose tissue oxidative stress

Lipolysis is a pro-oxidant event as triglyceride hydrolysis and the release of FA increase mitochondrial respiration and promote the production of reactive oxygen species (ROS) [38]. In this group of cows, periparturient lipolysis led to the activation of pathways regulating responses to ROS and oxidative stress (Fig. 3E). It is important to emphasize the postpartum enhancement of the transcription of genes related to heme processing, including *HMOX1*, *HP*, and *STEAP2.* Heme oxygenase 1 is encoded by *HMOX1* and is the rate-limiting enzyme of heme degradation that releases biliverdin, ferritin, and carbon monoxide [63]. As for *STEAP2,* this gene encodes the metalloprotease STAMP1 that reduces iron [64]. In AT, haptoglobin (encoded by *HP*) exerts an antioxidant role, and its expression is triggered by inflammatory responses such as those induced by lipolysis [65]. Heme induces lipolysis in adipocytes through a mechanism that involves the generation of pro-oxidants and lipid peroxidation products [63]. Importantly, IPA analyses of our data identified *HMOX1* together with C3 as possible upstream determinants in the production of ROS (Fig. 5c). Also, IPA analysis identified carbon monoxide as a link between heme processing and macrophage infiltration. Carbon monoxide is the product of heme oxidase 1 and is a promoter of maturation of monocytes into macrophages as it induces the expression of CD14 [66]. Taken together, the transcriptomic patterns of heme processing proteins and carbon monoxide indicate that in healthy PPE cows, as those in the present study, the AT rapidly deploys antioxidant defenses to reduce the pro-lipolytic effects of heme and other inducers of oxidative stress and at the same time trigger the rapid resolution of inflammation by enhancing the maturation of infiltrating monocytes.

In our present study, DEG, pathway, and IPA analyses identified the nuclear receptor *VDR as* a key target gene that is involved in several upregulated processes post-calving. Vitamin D exerts many of its effects through the activation of VDR. These include, but are not limited to, inhibiting lipolysis, activating lipogenesis and adipogenesis, minimizing adipocyte apoptosis, promoting adiponectin secretion, and exerting an anti-inflammatory effect in AT (reviewed extensively in [67]). The role that VDR plays in the adaptation of AT to PPE metabolic challenges is unknown in dairy cows; therefore, further research is warranted especially given the allelic variations in coding regions of the bovine *VDR* gene [68].

Finally, it is important to note that this study focused on AT and did not include samples from the liver, mammary gland, uterus, or other organs with significant physiological changes during PPE. Therefore establishing the exact mechanisms that drive specific AT responses during PPE based on the data presented is difficult. For example, systemic inflammation and uterine involution can also induce AT inflammatory responses directly and therefore enhance the lipolysis induced inflammation.

## Conclusion

Our present study presents a transcriptomic analysis that identified gene networks, processes, and pathways that orchestrate the adaptations of AT to the lipolytic responses that characterize the PPE in dairy cows. Results provide a comprehensive characterization of the gene expression dynamics at 1 and 2 weeks postpartum compared to a sample collected 1 week before parturition. DEG and pathway enrichment analyses identified important gene targets for periparturient AT processes such as lipid mobilization (*ABCA1, FASN, ELOV6*), inflammation (*C3, C5*, *CCL2 IL6*, *CXCL2*, and *TREM2*) extracellular matrix remodeling (*ADAMTS4* and *ADAM12*), and redox signaling (*HMOX1*, *HP*, *STEAP2,* and VDR) and demonstrate the complex interactions among these essential functions during PPE.

## Supplementary Information

**Additional file 1: Table S1**. Ingredient and nutrient composition of the close-up diet and the postpartum diet.

**Additional file 2.**

**Additional file 3.**

## Data Availability

Raw data generated and used during this study are included in this published article and its supplementary information files. Sequencing data from this study has been deposited in the National Center for Biotechnology Information Gene Expression Omnibus (GEO) and is accessible through the GEO Series accession number GSE159224. All other relevant data are available within the article and supplement files or from the corresponding author upon request.

## Abbreviations

AT: Adipose tissue
BCS: Body condition score
BHB: Beta hydroxybutyrate
BW: Body weight
FA: Fatty acids
FFA: Free fatty acids
LPS: Lipopolysaccharide
NEB: Negative energy balance
NEFA: Non-esterified fatty acids
PPE: Periparturient period
SCAT: Subcutaneous adipose tissue
