# Supplementary material for: Transcriptomic profiling of adipose tissue inflammation, remodeling, and lipid metabolism in periparturient dairy cows (Bos taurus)
Source: BMC Genomics. 2020 Nov 23;21:824. doi: 10.1186/s12864-020-07235-0 (PMC7686742; doi:10.1186/s12864-020-07235-0)
Supplement: Supplementary file 1 — Additional file 1: Table S1. Ingredient and nutrient composition of the close-up diet and the postpartum diet. [file 12864_2020_7235_MOESM1_ESM.docx]

| **Item** | **Diet** | |
| --- | --- | --- |
|  | **Close-up** | **Fresh** |
| ***Ingredient, % DM*** |  |  |
| Corn Silage | 28.0 | 30.0 |
| Grass Hay | 32.2 | - |
| Alfalfa Hay | - | 18.1 |
| Alfalfa Silage | - | 11.7 |
| Soybean Meal | 9.6 | 9.7 |
| Ground Corn | 6.6 | 15.0 |
| Soyhulls | - | 5.6 |
| High Moisture Corn | - | 4.4 |
| Mineral and vitamin mix^1,2^ | 8.5 | 4.0 |
| Fatty Acid Supplement^3^ | 1.2 | 1.6 |
| ***Nutrient Composition, % DM^4^*** |  |  |
| NDF | 42.5 | 32.5 |
| Forage NDF | 95.3 | 27.5 |
| iNDF | 18.0 | 14.2 |
| CP |  | 17.5 |
| Starch |  | 25.9 |
| FA | 1.2 | 1.6 |
| FA g/100g FA |  |  |
| 16:0 | 18,2 | 17.1 |
| 18:0 | 3.6 | 3.4 |
| *cis-9* 18:1 | 19.8 | 15.1 |
| *cis-9, cis-12* 18:2 | 49.7 | 48.8 |
| *cis-9, cis-12, cis-15* 18:3 | 4.9 | 8.5 |

Supplement Table 1: Ingredient and nutrient composition of the close-up diet and the postpartum diet.

^1^ Vitamin-mineral mix for the close-up diet contained (DM basis): 54.8% SoyChlor, 13.9% limestone, 10.0% rumen-protected choline, 8.8% di- calcium phosphate, 4.2% magnesium sulfate, 1.8% salt, 1.8% yeast, 4.4% trace minerals and vitamins, and 0.3% selenium yeast 600 (600 mg of Se/kg).

^2^ Vitamin-mineral mix for the fresh cow diet (DM basis): 27.9% molasses, 15.3% limestone, 12.2% sodium bicarbonate, 11.8% blood meal, 8.7% dicalcium phosphate, 6.1% trace minerals and vitamins, 5.7% rumen-protected choline, 4.4% magnesium sulfate, 3.9% salt, 2.7% animal fat, 0.9% yeast, and 0.4% selenium yeast 600 (600 mg of Se/kg).

^3^ Palmitic acid-enriched FA supplement (Nutracor; Wawasan Agrolipids, Johor, Malaysia). The supplement contained (g/100 g of fatty acid) 0.64 of C14:0, 84.5 of C16:0, 1.80 of C18:0, 7.88 of C18:1 *cis*-9, and 99.0% total fatty acids. Blend of Ca salts of palm FA supplement (Nutracal; Wawasan Agrolipids, Johor, Malaysia) and Palmitic acid-enriched FA supplement (Nutracor; Wawasan Agrolipids, Johor, Malaysia).

^4^ Expressed as percent of as fed.
